# 

**Published:** 1893-03

**Authors:** 


### CONTENTS.

ORIGINAL ARTICLES—The Management of Suppuration, Complicating Tubuculous Disease of the Bones and Joint, by V. P. Gibney, M. D., New York........ 111
The Pathology of Carcinoma, by H. C. Coe, M. D. New York........ 121
Clinical Reports, of W. T. Bull, M. D., New York........ 123
CORRESPONDENCE—Our New York Letter........ 130
Our London Letter........ 132
SOCIETY REPORTS—Clinical Society of Maryland........ 134
BOOK NOTICES—A Pocket Medical Dictionary, by George M. Gould; A Treatise on Disease of the Rectum, Anus and Sigmoid Flexure, by J. M. Matthews, M. D.; Electro-Therapeutics of Gynecology, by A. H. Goelet, M. D........145–46
SELECTIONS AND ABSTRACTS—The Operative Treatment of Chronic Glaucoma........ 144
Female, to Nervous and Mental Diseases........ 147
A New Sign in the Diagnosis of Intra-Cranial Affections........ 148
Papoid........ 149
Nocturnal Incontinence of Urine and Phimosis........ 150
Acute Tonsilitis........ 151
Uterine Tampon........ 151
EDITORIAL—Georgia Medical Association........ 152
English as it is read in the Grady Hospital........ 159
Commencement Southern Medical College........ 153
Medical Department of the University........ 154
Commencement Atlanta Medical College........ 155
EDITORIAL NOTES........157–58
BOOKS AND PAMPHLETS RECEIVED........ 159
PRESCRIPTIONS........160–61
SPECIAL NOTES........162–65

